# Nonlinear Optics in Low-Dimensional Nanomaterials

**DOI:** 10.3390/nano15151191

**Published:** 2025-08-03

**Authors:** Dawei Li, Mengmeng Wang

**Affiliations:** 1 School of Optoelectronic Engineering and Instrumentation Science, Dalian University of Technology, Dalian 116024, China; 2 Laser Micro/Nano Fabrication Laboratory, School of Mechanical Engineering, Beijing Institute of Technology, Beijing 100081, China

## 1. Introduction

Nonlinear optics describes the study of optical phenomena related to the interaction between intense light fields and matter [1]. Conventional nonlinear optical devices are based on bulk materials. However, these nonlinear materials have certain limitations, such as a low nonlinear susceptibility, large size dimension, and phase matching issues, that make them unsuitable for the future development of compact, integrated nonlinear optical devices [2]. Recent investigations have shown that a wide variety of low-dimensional materials can provide strong nonlinear optical responses compared to bulk nonlinear materials [3]. Thus, nonlinear optics in low-dimensional materials have become an active research field [4,5,6,7,8,9,10], making them a versatile playground for exploring ultrafast light–matter interactions and developing nonlinear nanophotonic and optoelectronic applications that range from wavelength conversion and ultrashort laser pulse generation to quantum photonics and integrated photonics [11,12,13,14].

This Special Issue aims to present the latest experimental and theoretical research on nonlinear optics in various low-dimensional materials (LDMs) and LDM-based nonlinear optical applications. Finally, this Special Issue collected ten articles, including seven research articles [15,16,17,18,19,20,21] and three review papers [22,23,24].

## 2. An Overview of the Published Articles

In Contribution 1, Kong et al. reported a facile, low-cost strategy for fabricating wafer-scale surface-enhanced Raman scattering (SERS) substrates based on Ag-TiO_2_ nanoparticle–film hybrids by combining dip-coating and UV light array photo-deposition [15]. The large-scale AgNP/TiO_2_ hybrids working as SERS substrates show high sensitivity and good uniformity at both the micron and wafer levels. The as-prepared flexible AgNP/TiO_2_ SERS substrate is mechanically robust and shows excellent performance in detecting sub-micrometer-sized plastics and low-concentration organic pollutants on complex surfaces.

In Contribution 2, Murawski et al. characterized the inter-band cascade long-wave infrared (LWIR) detector with a type-II InAs/InAsSb superlattice absorber via photoluminescence (PL) and spectral response (SR) methods [16]. They grew InAs/InAsSb heterostructures via molecular beam epitaxy on a GaAs substrate (001) orientation, with the crystallographic quality confirmed via high-resolution X-ray diffraction. They determined the transitions between the superlattice minibands by combining PL, SR, and theoretical calculations.

In Contribution 3, Rashid et al. presented a simple scheme to simultaneously grow distinct geometries of cesium lead bromide microcrystals, including microrods, microplates, and microspheres, in a single chemical vapor deposition experiment [17]. Structural analysis revealed high crystallinity and an orthorhombic phase of the as-grown perovskite microcrystals. Moreover, the nonlinear optical characteristics of the as-grown perovskite microcrystals were validated through low-threshold photonic lasing, demonstrating their potential for future optoelectronic applications.

In Contribution 4, Fan et al. explored the asymmetry observed between the effects of photon–phonon coupling and crystal fields (CFs) on the fine structure of fluorescence and spontaneous four-wave mixing (SFWM) in Eu^3+^: BiPO_4_ and Eu^3+^: NaYF_4_ microcrystals [18]. The competition between the CF and the photon–phonon dressing leads to dynamic splitting in two directions. The CF leads to static splitting in one direction under weak phonon dressing. The evolution from strong dressing to weak dressing results in spectral asymmetry. This spectral asymmetry includes out-of-phase fluorescence and in-phase SFWM.

In Contribution 5, Klenen et al. studied the tuning of second and third harmonic emission in mechanochemically synthesized nanocrystalline LiNb_1−*x*_Ta_*x*_O_3_ (0 ≤ *x* ≤ 1) using nonlinear diffuse femtosecond-pulse reflectometry with wavelength-tunable fundamental femtosecond laser pulses from 1200 nm to 2000 nm [19]. They validated a gap-free harmonic emission that agreed with the theoretically expected energy relations, dependencies on intensity and wavelength, as well as spectral bandwidths for harmonic generation.

In Contribution 6, Liu et al. reported the in vivo, simultaneous 3D labeling and imaging of potato cell structures using plasmonic nanoprobe-assisted multimodal nonlinear optical microscopy [20]. They found that the complete cell structure could be imaged via the combination of second-harmonic generation (SHG) and two-photon luminescence (TPL) when noble metal silver or gold ions were added. During the SHG-TPL imaging process, noble metal ions that crossed the cell wall were rapidly reduced to plasmonic nanoparticles with the femtosecond laser and selectively anchored onto both sides of the cell wall, thereby leading to the simultaneous 3D labeling and imaging of the potato cells.

In Contribution 7, Bundulis et al. characterized the nonlinear optical properties of indium–tin oxide (ITO) quantum dots (QDs) in the near-infrared range [21]. They presented the results of three-photon absorption (TPA), third-harmonic generation (THG), and Kerr-effect-induced nonlinear refraction in ITO QDs. The Kerr-induced nonlinear refractive index showed an increase from −6.7 × 10^−18^ to −1.5 × 10^−17^ m^2^ W^−1^ along the 800–950 nm range. The TPA coefficient at 950 nm was measured to be 1.42 × 10^−25^ m^3^/W^2^. The absolute value of THG conversion efficiency in the thin film at 1350 nm was estimated to be ~10^–5^.

In Contribution 8, Lu et al. briefly reviewed the developmental progress of laser-induced graphene (LIG)-based electromagnetic stealth materials, with a particular emphasis on doping technologies and shielding mechanisms tailored for stealth applications [22]. Furthermore, they proposed potential future development pathways for LIG-based stealth materials to facilitate their transition toward broader practical applications.

In Contribution 9, Xin et al. outlined the principles of nonlinear optics and the magnetic structures of two-dimensional (2D) materials, reviewed recent progress in nonlinear optical studies, including magnetic structure detection and nonlinear optical imaging, and highlighted their role in probing magnetic properties by combining SHG and multispectral integration [23]. Finally, they discussed the prospects and challenges in applying nonlinear optics to 2D magnetic materials, emphasizing their potential in next-generation photonic and spintronic devices.

In Contribution 10, Fu et al. provided a thorough review of recent advancements in the investigation of highly efficient and adjustable SHG processes in LDMs [24]. They succinctly introduced the fundamental theory bases of SHG for LDMs and the experimental method for second-order nonlinear LDMs. In addition, they systematically reviewed the modulation of SHG response strategies in LDMs and the development of multifunctional and practical LDMs in optical characterizations and applications. Finally, they discussed the current state and challenges of SHG for LDMs, as well as their practical applications in nonlinear integrated devices.

## 3. Conclusions

In conclusion, this Special Issue compiles ten original research articles and review papers. These contributions may promote the development of low-dimensional nonlinear optical materials and their practical applications in nanophotonics and optoelectronics. Furthermore, this Special Issue is expected to be of interest to a broader audience in the field of low-dimensional optical nanomaterials.

## References

[1] Boyd R.W., Gaeta A.L., Giese E., Drake G.W.F. (2023). Nonlinear Optics. Springer Handbook of Atomic, Molecular, and Optical Physics.

[2] Armstrong J.A., Bloembergen N., Ducuing J., Pershan P.S. (1962). Interactions Between Light Waves in a Nonlinear Dielectric. Phys. Rev..

[3] Yamashita S. (2018). Nonlinear Optics in Carbon Nanotube, Graphene, and Related 2D Materials. APL Photonics.

[4] Li D.W., Zhou Y.S., Huang X., Jiang L., Silvain J.F., Lu Y.F. (2015). In Situ Imaging and Control of Layer-by-Layer Femtosecond Laser Thinning of Graphene. Nanoscale.

[5] Li D., Xiong W., Jiang L., Xiao Z., Rabiee Golgir H., Wang M., Huang X., Zhou Y., Lin Z., Song J. (2016). Multimodal Nonlinear Optical Imaging of MoS_2_ and MoS_2_-Based van der Waals Heterostructures. ACS Nano.

[6] Li D., Wei C., Song J., Huang X., Wang F., Liu K., Xiong W., Hong X., Cui B., Feng A. (2019). Anisotropic Enhancement of Second-Harmonic Generation in Monolayer and Bilayer MoS_2_ by Integrating with TiO_2_ Nanowires. Nano Lett..

[7] Wang M., Li D., Liu K., Guo Q., Wang S., Li X. (2020). Nonlinear Optical Imaging, Precise Layer Thinning, and Phase Engineering in MoTe_2_ with Femtosecond Laser. ACS Nano.

[8] Li D., Huang X., Xiao Z., Chen H., Zhang L., Hao Y., Song J., Shao D.-F., Tsymbal E.Y., Lu Y. (2020). Polar Coupling Enabled Nonlinear Optical Filtering at MoS_2_/Ferroelectric Heterointerfaces. Nat. Commun..

[9] Li D., Huang X., Wu Q., Zhang L., Lu Y., Hong X. (2023). Ferroelectric Domain Control of Nonlinear Light Polarization in MoS_2_ via PbZr_0.2_Ti_0.8_O_3_ Thin Films and Free-Standing Membranes. Adv. Mater..

[10] Li D., Hou X., Kong F., Wang K., Hong X. (2024). Giant Modulation of Second-Harmonic Generation in CuInP_2_S_6_ by Interfacing with MoS_2_ Atomic Layers. ACS Nano.

[11] Gu L., Zhou Y. (2025). Nonlinear Optics in 2D Materials: From Classical to Quantum. Appl. Phys. Rev..

[12] Autere A., Jussila H., Dai Y., Wang Y., Lipsanen H., Sun Z. (2018). Nonlinear Optics with 2D Layered Materials. Adv. Mater..

[13] Liu X., Guo Q., Qiu J. (2017). Emerging Low-Dimensional Materials for Nonlinear Optics and Ultrafast Photonics. Adv. Mater..

[14] Ryu H., Xu K., Li D., Hong X., Zhu W. (2020). Empowering 2D nanoelectronics via ferroelectricity. Appl. Phys. Lett..

[15] Kong F., Ji C., Zhao G., Zhang L., Hao Z., Wang H., Dai J., Huang H., Pan L., Li D. (2024). Controlled Fabrication of Wafer-Scale, Flexible Ag-TiO_2_ Nanoparticle–Film Hybrid Surface-Enhanced Raman Scattering Substrates for Sub-Micrometer Plastics Detection. Nanomaterials.

[16] Murawski K., Majkowycz K., Kopytko M., Manyk T., Dąbrowski K., Seredyński B., Kubiszyn Ł., Martyniuk P. (2024). Optical Characterization of the Interband Cascade LWIR Detectors with Type-II InAs/InAsSb Superlattice Absorber. Nanomaterials.

[17] Rashid M.U., Tahir Z., Sheeraz M., Ullah F., Park Y.C., Maqbool F., Kim Y.S. (2024). Controlled Morphological Growth and Photonic Lasing in Cesium Lead Bromide Microcrystals. Nanomaterials.

[18] Fan H., Zhang Z., Hussain I., Yang Q., Majeed M.K., Imran M., Raza F., Li P., Zhang Y. (2024). The Asymmetry Observed between the Effects of Photon–Phonon Coupling and Crystal Field on the Fine Structure of Fluorescence and Spontaneous Four-Wave Mixing in Ion-Doped Microcrystals. Nanomaterials.

[19] Klenen J., Sauerwein F., Vittadello L., Kömpe K., Hreb V., Sydorchuk V., Yakhnevych U., Sugak D., Vasylechko L., Imlau M. (2024). Gap-Free Tuning of Second and Third Harmonic Generation in Mechanochemically Synthesized Nanocrystalline LiNb_1−x_TaxO_3_ (0 ≤ x ≤ 1) Studied with Nonlinear Diffuse Femtosecond-Pulse Reflectometry. Nanomaterials.

[20] Liu K., Lei Y., Li D. (2023). Simultaneous 3D Construction and Imaging of Plant Cells Using Plasmonic Nanoprobe-Assisted Multimodal Nonlinear Optical Microscopy. Nanomaterials.

[21] Bundulis A., Berzina A., Kim V.V., Polyakov B., Novikovs A., Ganeev R.A. (2023). Variation of Nonlinear Refraction and Three-Photon Absorption of Indium–Tin Oxide Quantum Dot Thin Films and Solutions in Near Infrared Range. Nanomaterials.

[22] Lu X., Su R., Chen G., Li W., Liang M., You R. (2025). Stealth Materials Based on Laser-Induced Graphene: Developments and Challenges. Nanomaterials.

[23] Xin Z., Xue B., Chang W., Zhang X., Shi J. (2025). Nonlinear Optics in Two-Dimensional Magnetic Materials: Advancements and Opportunities. Nanomaterials.

[24] Fu Y., Liu Z., Yue S., Zhang K., Wang R., Zhang Z. (2024). Optical Second Harmonic Generation of Low-Dimensional Semiconductor Materials. Nanomaterials.

